# Case Report: *Streptococcus suis*-associated meningoencephalitis with profound bilateral labyrinthine deafness and intra-operative cochlear fibrosis treated by cochlear implantation

**DOI:** 10.3389/fmed.2026.1941314

**Published:** 2026-09-25

**Authors:** Lingping Ding, Huan Wang, Fanghua Li

**Affiliations:** 1 Department of Otolaryngology–Head and Neck Surgery, West China Hospital, Sichuan University, Chengdu, China; 2 Department of Pathology, Sichuan Provincial People’s Hospital, University of Electronic Science and Technology of China, Chengdu, China

**Keywords:** bacterial meningoencephalitis, cochlear fibrosis, cochlear implantation, *Klebsiella pneumoniae*, labyrinthitis, metagenomic next-generation sequencing, *Streptococcus suis*, suspected additional bloodstream-borne *Klebsiella pneumoniae* infection

## Abstract

*Streptococcus suis* is a common zoonotic pathogen that usually causes monomicrobial meningitis. Occult bloodstream detection of *Klebsiella pneumoniae*, together with proven central-nervous-system *S. suis* infection, followed by irreversible cochlear fibrotic changes requiring cochlear implantation, appears exceptionally rare. We report a 52-year-old male with chronic heavy alcohol intake and occupational swine-exposure history who developed severe sepsis, recurrent suppurative meningoencephalitis caused by *S. suis*, and permanent bilateral labyrinthine-related deafness. Combined blood and cerebrospinal fluid (CSF) metagenomic next-generation sequencing (mNGS) identified suspected additional bloodstream infection by *K. pneumoniae*, although *K. pneumoniae* was not detected in CSF. Multimodal antimicrobial, anti-inflammatory, and immunomodulatory therapy cleared intracranial infection but failed to restore hearing. Preoperative imaging suggested cochlear fibrotic changes; left-sided cochlear implantation achieved full electrode insertion with stable intraoperative neural responses despite abundant intracochlear fibrous tissue observed intraoperatively. This case raises three important considerations: occupational swine exposure together with chronic alcoholism may plausibly contribute to suspected polymicrobial sepsis; in selected patients, peripheral-blood mNGS may help identify occult Gram-negative bacteremia when CSF assays remain negative during recurrent meningitis; and cochlear fibrosis may not absolutely preclude technically-successful cochlear implantation.

## Introduction

*Streptococcus suis* is a zoonotic pathogen that typically causes monomicrobial meningitis, among which sensorineural hearing loss represents a frequent, potentially permanent sequela, occurring in a substantial proportion of survivors (1). Post-meningitic inner-ear inflammation may further progress to cochlear fibrosis or ossification, which increases technical difficulty for subsequent cochlear implantation. Meningoencephalitis due to S. suis accompanied by suspected additional bloodstream-borne Klebsiella pneumoniae (without CSF evidence for K. pneumoniae CNS invasion), progressing to profound deafness requiring cochlear implantation, appears extraordinarily rare (2). For patients with irreversible profound sensorineural hearing loss secondary to bacterial meningitis, cochlear implantation constitutes the primary rehabilitative option; however, optimal surgical timing in the setting of intra-cochlear fibrotic change remains debated (3, 4). Here we describe a case that illustrates the diagnostic challenges and therapeutic strategies for this complex condition.

## Case presentation

### Initial presentation and critical care (January–March 2026)

The patient was a 52-year-old male with a 10-year smoking history, chronic daily alcohol consumption of approximately 100 g, and occupational swine-exposure history. Following heavy alcohol ingestion in January 2026, he developed vomiting, diarrhoea, progressive bilateral hearing impairment and vertigo. Upon initial hospital admission, the patient deteriorated rapidly and progressed to septic shock complicated by multiple-organ dysfunction. Blood culture yielded *S. suis* with clindamycin resistance; CSF culture and mNGS confirmed *S. suis* meningoencephalitis (5). He initially received meropenem and clindamycin. After *S. suis* with in-vitro clindamycin resistance was isolated from blood culture, clindamycin was temporarily withdrawn and replaced by linezolid. Following multidisciplinary consultation, clindamycin was subsequently reintroduced in combination with meropenem: despite in-vitro resistance, clindamycin may suppress streptococcal exotoxin/superantigen synthesis, an adjunctive benefit independent of bactericidal activity (6). Intravenous immunoglobulin and tapered corticosteroids were also administered (7); nevertheless, his hearing continued to decline.

On February 23, recurrent high-grade fever and elevated inflammatory markers developed. Repeated CSF mNGS detected no pathogens, but peripheral-blood mNGS uniquely identified *K. pneumoniae*, indicating a suspected additional bloodstream infection; *K. pneumoniae* was not recovered from CSF specimens (8). Ceftazidime-avibactam was added to the antimicrobial regimen, and the patient was transferred to our center for further care.

### Inpatient evaluation and management (March 3–30, 2026)

Upon admission to our hospital, temporal-bone MRI demonstrated diffuse bilateral labyrinthine enhancement suggestive of labyrinthitic injury. Definite radiological evidence for bilateral cochlear fibrosis was limited; more prominent intra-cochlear signal abnormalities were predominantly observed on the right-sided MRI sequences. MRI findings were therefore suggestive rather than confirmatory of cochlear fibrosis, whereas intracochlear fibrous tissue was directly confirmed intraoperatively only within the left cochlea during implantation. Auditory brainstem response verified bilateral profound sensorineural hearing loss. Serial CSF assessments demonstrated persistently elevated CSF protein despite declining nucleated-cell counts. Persistent CSF protein elevation may reflect residual central-nervous-system inflammation or blood-CSF-barrier dysfunction, though concurrent chronic inner-ear inflammatory injury cannot be excluded (Table 1) (9). Repeated lumbar punctures revealed that CSF nucleated-cell counts normalized by March 18, while protein concentrations remained elevated. The patient was discharged home on oral suppressive antibiotic therapy on March 30.

**Table 1:** Serial cerebrospinal fluid, microbiological and selected peripheral-blood laboratory findings.

Sampling date	Specimen	Microbiological findings	CSF Nucleated cells (×10⁶/L)	CSF microprotein (g/L)	Blood WBC (×10⁹/L)	PCT (ng/mL)	Clinical significance
2026-01-23	Blood+CSF	Blood culture: *S. suis* (clindamycin-resistant); CSF culture: *S. suis*; CSF mNGS: *S. suis*	Markedly elevated	Elevated	–	–	Initial proven *S. suis* meningoencephalitis
2026-02-23	Blood+CSF	Not performed on this date	961	0.56	2.13	<0.05	Recurrent high-grade fever with active CNS inflammation
2026-02-27	Blood+CSF	CSF mNGS: negative (bacteria, viruses, fungi); Blood mNGS: *K. pneumoniae* detected (blood culture not performed)	–	–	–	–	CSF negative for *K. pneumoniae*; suspected additional bloodstream infection by *K. pneumoniae*
2026-03-03	Blood	Not performed on this date	–	–	11.92	0.12	Admission baseline; persistent systemic inflammation
2026-03-09	CSF	Not performed on this date	120	0.73	–	–	Persistent CNS inflammation; incomplete resolution
2026-03-11	Blood	Not performed on this date	–	–	–	0.047	Declining procalcitonin, improved systemic inflammatory status
2026-03-18	CSF	CSF culture: negative (including fungal culture)	2	0.78	–	–	CSF cell count normalized; persistent protein elevation suggests residual CNS or blood-CSF-barrier disturbance

Cerebrospinal fluid (CSF) parameters (nucleated cells, microprotein) were measured from CSF samples. Blood WBC and procalcitonin (PCT) were measured from peripheral-blood samples collected on the indicated sampling dates; CSF was not tested for WBC or PCT. Microbiological methods are specified for each finding (blood culture, blood mNGS, CSF culture, CSF mNGS); “not performed” indicates that the corresponding assay was not conducted on that sampling date. A single high-sensitivity C-reactive protein value (hs-CRP, 150.33 mg/L) was available on 2026-02-23 and was not incorporated into this table due to lack of serial time-point data.

### Cochlear implantation (June 2026)

After three-month outpatient follow-up confirmed permanent deafness unresponsive to acoustic amplification, left-sided cochlear implantation was performed on June 8, 2026. Although MRI signal alterations appeared more marked on the right cochlea, bilateral profound sensorineural hearing loss was present; preoperative radiological assessment suggested more favourable middle-ear and mastoid anatomical conditions on the left side, and shared clinical decision-making favoured left-sided implantation. Intraoperatively, abundant fibrous tissue was identified within the left-ear scala tympani. Technically-successful full-array electrode insertion was accomplished with normal impedance and robust intraoperative electrically evoked compound action potentials. Post-operative thin-section temporal-bone CT verified satisfactory electrode position (Figure 1). No peri-operative complications occurred; the patient was discharged on postoperative day 3. Auditory functional rehabilitation outcomes remain pending scheduled device activation and follow-up assessments. Key diagnostic and therapeutic milestones throughout the disease course are summarized in the CARE-compliant timeline (Figure 2).

**Figure 1:**
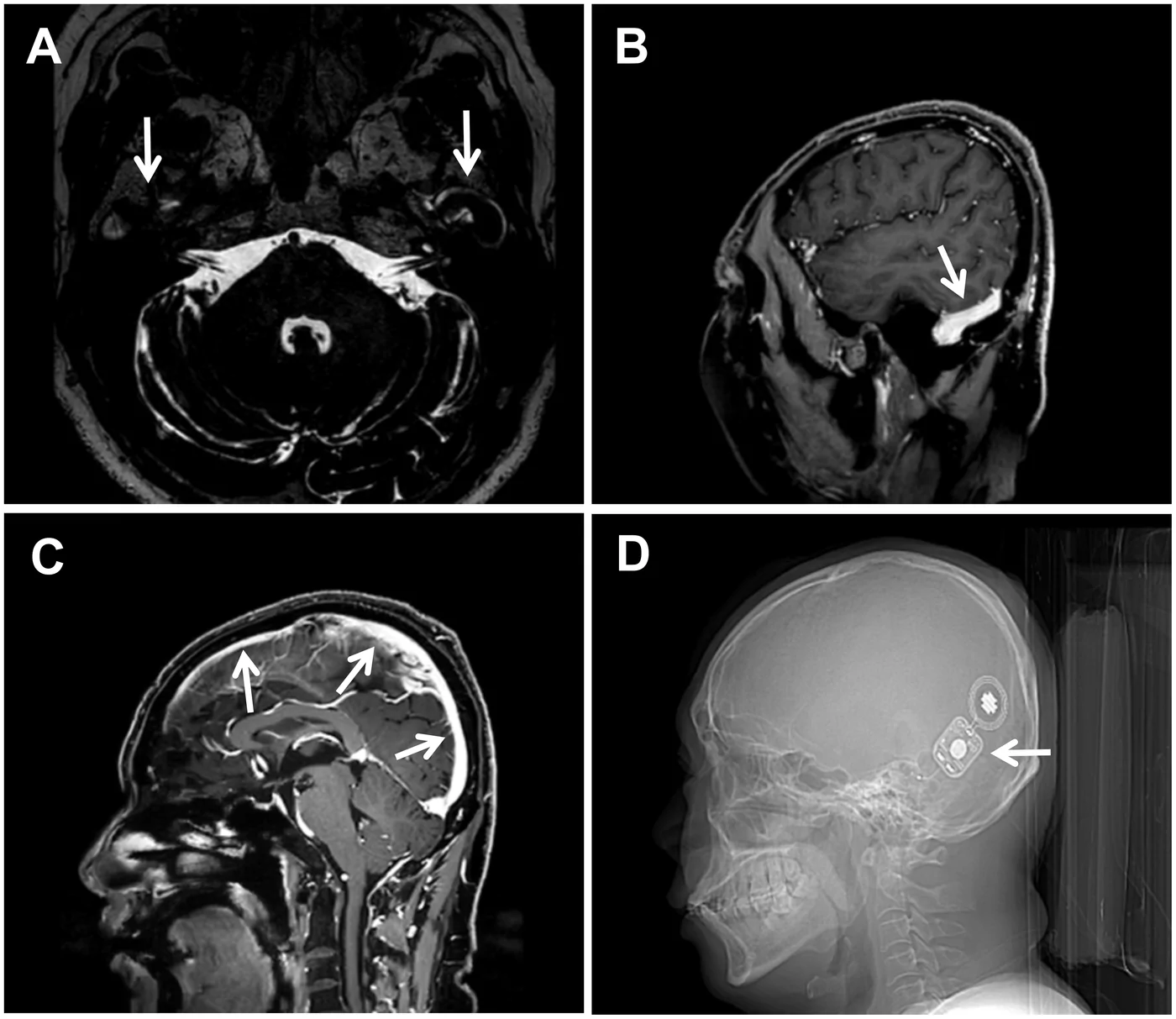
Preoperative MRI and postoperative CT. **(A)** Contrast-enhanced cranial MRI demonstrating bilateral labyrinthine enhancement (arrows), consistent with inflammatory labyrinthitis. **(B)** Thin-section temporal-bone post-contrast MRI showing intracochlear abnormal signal suggestive of cochlear fibrosis (arrow). Dedicated heavily-T2-weighted inner-ear sequences were not acquired during this clinical work-up and are therefore unavailable for this case. **(C)** Brain contrast-enhanced MRI showing meningeal enhancement and frontal-lobe encephalomalacia sequelae after meningoencephalitis. **(D)** Post-operative thin-section temporal-bone CT showing full electrode array placement within the fibrotic left cochlea after cochlear implantation.

**Figure 2:**
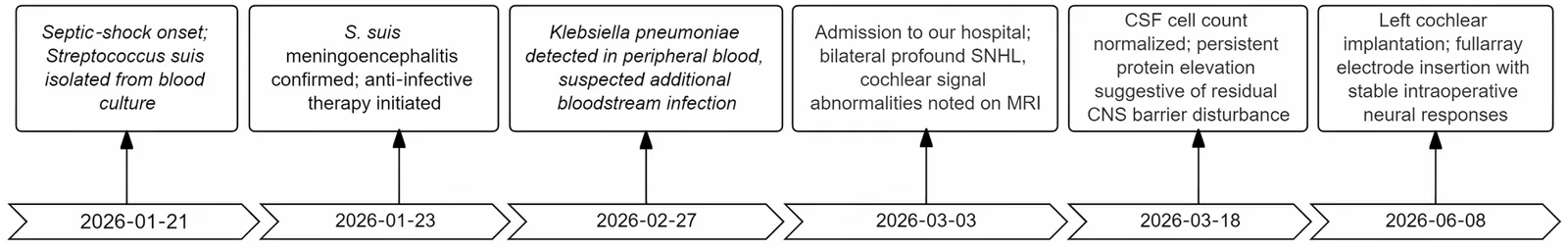
CARE-compliant timeline of key clinical events in this case.

### Differential diagnosis

In this patient presenting with recurrent *S. suis*-associated meningoencephalitis and permanent bilateral profound sensorineural hearing loss secondary to suspected bacterial labyrinthitis, alternative etiologies were considered. Clinical distinction between relapse of the original bacterial infection and de-novo reinfection can be challenging given overlapping clinical manifestations. Autoimmune labyrinthitis and viral labyrinthitis may also induce vertigo and profound hearing loss; however, the patient had clear microbiological evidence for bacterial infection, without serological or molecular evidence supporting autoimmune or viral etiology. A skull-base bony defect predisposing to recurrent meningitis was also considered; temporal-bone CT and MRI did not identify bony dehiscence or defect in this case. Taken together, clinical, laboratory and imaging findings supported a diagnosis of *S. suis*-mediated bacterial meningoencephalitis complicated by bacterial labyrinthitis, with suspected additional peripheral bloodstream-borne *K. pneumoniae* detection.

## Discussion

### Synergistic risk factors: occupational exposure and alcoholism

Most reported *S. suis* infections are monomicrobial (1, 10). In this patient, occupational swine contact and chronic heavy alcohol intake may plausibly act synergistically in disease progression, though this remains hypothetical for this single case. Alcohol impairs intestinal mucosal integrity and innate immunity, which raises the biological hypothesis that alcohol-related injury could facilitate *S. suis* translocation across gut epithelium and promote intestinal dysbiosis favouring *K. pneumoniae* translocation into the bloodstream (11, 12). Combined release of Gram-positive and Gram-negative toxins could theoretically amplify the systemic inflammatory response, potentially contributing to recurrent meningeal seeding and protracted clinical course (13). For febrile patients with swine-exposure history and chronic alcohol overuse, dual blood-CSF mNGS may be considered to reduce risk of missed polymicrobial infection (8, 14).

### Role of metagenomic next-generation sequencing

In this clinical scenario, peripheral-blood mNGS identified *K. pneumoniae* while CSF pathogen assays remained negative, suggesting a complementary diagnostic role for blood-based mNGS in selected patients with recurrent meningitis. High cerebrospinal-fluid antibiotic concentrations achieved via systemic intravenous administration may transiently suppress bacterial DNA detectable within CSF. The hypothesis that persistent blood-borne *K. pneumoniae* might potentially reseed the meninges through a damaged blood-brain barrier cannot be confirmed from this single-case observation (8, 15). Thus, blood mNGS may be considered for patients with recurrent meningitis and sterile CSF cultures to assist clinical decision-making for antimicrobial adjustment (8, 16).

### Rationale for antimicrobial and immunomodulatory therapies

Antimicrobial management in this case was adjusted repeatedly according to clinical course, microbiological results and local infectious-disease specialist consultation. Initial blood culture isolated *S. suis*, demonstrating in-vitro resistance to clindamycin. After multidisciplinary consultation, clindamycin was reintroduced despite phenotypic resistance. For Gram-positive streptococcal infections, clindamycin may suppress bacterial exotoxin/superantigen protein synthesis, a potential adjunctive benefit independent of bactericidal activity, even under conditions of *in-vitro* resistance (6). Importantly, clindamycin was always administered together with meropenem, which provided the principal bactericidal activity; clindamycin served strictly as an adjunctive agent rather than the primary therapeutic component. Meropenem was maintained for broad-spectrum central-nervous-system penetration. Intravenous immunoglobulin and tapered corticosteroids were administered during the acute severe phase, following institutional practice for severe streptococcal toxic-shock-like syndrome (7). When recurrent fever occurred and peripheral-blood mNGS identified *Klebsiella pneumoniae*, ceftazidime-avibactam was added to target this putative bloodstream pathogen. Antibiotic selection, dose adjustment and treatment duration were guided by serial clinical assessment, inflammatory biomarkers, and repeated cerebrospinal-fluid evaluations.

### Cochlear implantation in the fibrotic cochlea

Chronic post-meningitic labyrinthitis may lead to irreversible cochlear fibrosis, which can destroy hair cells and spiral ganglion neurons (17). Historically, intracochlear fibrous tissue was considered a relative contraindication to implantation because of concerns about electrode insertion difficulty and reduced neural stimulation. Our intraoperative findings (full-array insertion with normal impedances and robust intraoperative neural responses) suggest that intra-cochlear fibrosis alone may not absolutely preclude technically-successful implantation (3, 18). Recent case series have confirmed that cochlear implantation can be performed safely in patients with post-meningitic ossification or fibrosis; meaningful auditory rehabilitation outcomes still require individual postoperative follow-up assessment (4, 19). Therefore, imaging-suggested cochlear fibrotic change should not automatically serve as an absolute contraindication for surgical candidacy.

### Pathogen synergy and disease chronicity

The protracted clinical course raises the hypothesis of pathogen-related synergistic inflammatory effects: *S. suis* pyrogenic exotoxins may induce meningeal edema, while *K. pneumoniae* lipopolysaccharides could sustain pro-inflammatory cytokine release (13, 20). Even after CSF sterilization, the theoretical possibility that persistent bacteremia might potentially re-initiate meningeal inflammation over months cannot be proven in this single-case setting (21). Acute labyrinthitis may cause reversible oedema, but chronic inflammation can generate fibrous scarring that blocks auditory signal transmission; under such circumstances, cochlear implantation may represent the primary available rehabilitative option (17, 22).

Compared with previously-reported cases of *Streptococcus suis*-associated meningoencephalitis, most published reports describe monomicrobial infection (1, 2). This case is distinguished by proven *S. suis* central-nervous-system infection with suspected additional bloodstream-borne *K. pneumoniae*, clinically apparent bilateral profound sensorineural hearing loss, intra-operative identification of left-sided cochlear fibrosis, and technically-successful cochlear implantation (3). Detailed quantitative comparison with cumulative published cases will be pursued in future work.

## Conclusion

*S. suis*-mediated central-nervous-system infection with suspected additional bloodstream-borne *K. pneumoniae* represents a rare clinical scenario associated with occupational swine exposure and heavy alcohol consumption. Persistent intracranial inflammatory injury may result in bilateral labyrinthitic changes and intra-cochlear fibrotic alterations. Combined blood-CSF mNGS facilitates pathogen identification for complex infectious presentations. Technically-successful cochlear implantation is feasible for permanent post-meningitic deafness even in the setting of imaging-suggested intra-cochlear fibrosis; functional auditory outcomes await post-device-activation clinical follow-up.

### Limitations

This report is based on a single-patient case and therefore cannot establish definitive causal relationships. Mechanisms concerning bacterial translocation, toxin-mediated inflammation and pathogen synergism remain speculative, consistent with the constraints of case-report methodology. Post-operative functional auditory rehabilitation outcomes including Categories of Auditory Performance (CAP), Speech Intelligibility Rating (SIR), speech-perception scores, aided pure-tone average (PTA), and patient-reported satisfaction remain unavailable at the time of manuscript submission, as formal assessments require device activation and longitudinal rehabilitation. Only intraoperative electrophysiological data are presented in the current report. The diagnostic and therapeutic approaches described reflect management for this individual patient and should not be generalized as universal clinical guidelines. While multimodal testing was performed, we cannot fully exclude rare alternative underlying causes for labyrinthine injury. Further cumulative case data are required to validate our observations.

## Data Availability

The raw metagenomic sequencing datasets are not publicly available to protect patient privacy and comply with hospital-ethical regulations. All de-identified findings supporting the conclusions of this article are included within the manuscript. Qualified researchers may request relevant de-identified raw data from the corresponding author upon formal institutional application.
